# Ankle exoskeleton torque controllers based on soleus muscle models

**DOI:** 10.1371/journal.pone.0281944

**Published:** 2023-02-27

**Authors:** Paul S. Pridham, Leia Stirling

**Affiliations:** 1 Department of Aeronautics and Astronautics, Massachusetts Institute of Technology, Cambridge, MA, United States of America; 2 Industrial and Operations Engineering, Robotics Institute, University of Michigan, Ann Arbor, MI, United States of America; University of Illinois at Urbana-Champaign, UNITED STATES

## Abstract

Powered exoskeletons are typically task-specific, but to facilitate their wider adoption they should support a variety of tasks, which requires generalizeable controller designs. In this paper, we present two potential controllers for ankle exoskeletons based on soleus fascicles and Achilles tendon models. The methods use an estimate of the adenosine triphosphate hydrolysis rate of the soleus based on fascicle velocity. Models were evaluated using muscle dynamics from the literature, which were measured with ultrasound. We compare the simulated behavior of these methods against each other and to human-in-the-loop optimized torque profiles. Both methods generated distinct profiles for walking and running with speed variations. One of the approaches was more appropriate for walking, while the other approach estimated profiles similar to the literature for both walking and running. Human-in-the-loop methods require long optimizations to set parameters per individual for each specific task, the proposed methods can produce similar profiles, work across walking and running, and be implemented with body-worn sensors without requiring torque profile parameterization and optimization for every task. Future evaluations should examine how human behavior changes due to external assistance when using these control models.

## Introduction

Exoskeletons have the potential to enhance human function under a variety of conditions, with many individuals who would benefit from external assistance (e.g., the elderly, factory workers, search and rescue teams, military operators). Designing exoskeletons specialized for individual tasks results in mechanical designs, as well as controllers, which are highly tailored to their assigned task, thereby limiting their versatility. This paper will present two controllers that have the potential to provide personalized assistance across a variety of tasks, using active ankle exoskeleton control as a test case. The ankle is an area of particular interest for exoskeletal assistance, as it is a key contributor to propulsion in gait [1–3], and utilizes the Achilles tendon, which stores and returns energy over the gait cycle [4, 5]. The metabolic rate [6] of the soleus (a uni-articular plantar-flexor of the ankle) has been correlated with overall metabolic cost in locomotion [7]. These features and an existing body of literature in the exoskeleton field [8–11] make the ankle a strong candidate joint for evaluating new methods of exoskeleton control.

Researchers have previously used a variety of approaches for controller creation, including heuristics [11–13]; optimization for energy expenditure [8, 10, 14, 15], speed [16], or user preference [17]. Enabling controllers for a task or individual can take the form of refitting or tuning parameters, or completely changing the nature of the assistance provided depending on the method used. Task specific controller implementation on a single exoskeleton requires inferring the intended usage in real-time in order to appropriately transition between controllers to apply the appropriate assistance. The identification of tasks is an open area of research [18, 19], and adds an additional level of complexity to the system to support these control-mode transitions.

It would ultimately be preferable to develop a universal controller that is applicable across tasks, eliminating the complexities of mode switching or parameter selection. Development of a universal controller requires understanding the influencing factors, which depend on the goal of the exoskeleton. A common objective is the reduction of metabolic cost [9, 12, 13] and will be the focus of the controllers explored in this paper. The metabolic impact of exoskeleton use is affected by the energy used by the muscles moving the assisted joint, the energy used by the muscles at other joints, and the energy stored in connective tissue like the Achilles tendon [7]. To better understand the roles played by these factors, this paper will explore different controllers based on chemical energy used by the muscle.

Walking and running are useful first areas to explore for general controllers, while covering a large portion of the common locomotion types. In a typical walking or running cycle, the leg accepts the load in early stance when the foot strikes the ground, tensioning the Achilles tendon. Then the body moves in front of the foot, which further tensions the tendon. When the stance phase terminates with the foot pushing off the ground, the energy stored in the tendon is released, and the leg then swings through to restart the cycle. Initial exoskeleton controls designed to support gait explored mimicking the human ankle joint torque profile to assist the user [9, 20]. However, this direct method interfered with the utilization of the tendon as an energy storage element [9]. To address this drawback, researchers applied a torque profile similar to the positive power of the ankle joint [7, 12, 21]. Since tendon loading is generally associated with power absorption (negative power) at the ankle joint, this method is able to assist with push-off without interfering with the loading of the Achilles tendon in early stance. While this method has been effective at reducing the metabolic cost of walking [12, 15], it is heavily driven by the tendon’s passively returned power. However, the metabolic cost comes from the active energy used by the muscle, which is less represented in the overall mechanical power. By not accounting for this more costly form of energy usage, an important part of the system is being overlooked. There are approaches other than using joint power that account for how the body uses both the mechanical energy and chemical energy at a single joint or throughout the body, such as human-in-the-loop (HITL) optimization.

HITL optimization of assistive torque profiles accounts for hard-to-model aspects of the whole human-exoskeleton system, such as changes in muscle activation at other joints in response to the provided assistance. This method optimizes parameters of a general torque profile for an individual using an objective function, eliminating the assumptions that come from trying to apply a model to individuals with different physical characteristics [8, 10, 15]. However, it is a very time-intensive process, which does not generalize across tasks, and must be repeated for every activity the person may want to perform, as different tasks result in different torque profiles [8, 10]. Therefore, developing a single controller that can be used for a variety of tasks, transition between tasks, and be personalized for an individual requires a prior understanding of which biological factors drive the shape of these HITL curves, and the creation of models driven by these factors.

Recently, Slade et al. [22] attempted to generalize the HITL optimized model across various walking speeds by interpolating the parameters for the ankle torque profile used by Zhang et al. [8], when prior HITL optimization had occurred at three different speeds. They found that this speed-adaptive, estimated optimum (EO) model reduced metabolic cost on a treadmill when the speed was varied sinusoidally when compared to a generic fixed mode. They further compared this speed adaptive controller to one that had been individually optimized in real-world conditions and found that the individualized controllers had lower cost of transport than the generic speed-adaptive controller. These findings emphasize the importance of both adaptive and personalized controllers. However, this method still requires HITL optimization which requires data collection on the order of hours when using metabolic cost, and around half an hour when estimating relative metabolic cost, for a single speed/task.

In addition to the power and HITL-based approach, energy shaping has been used to create controllers that generalize across ground slopes [23]. Energy shaping works using the kinetic and potential energy of body segments so the controller can account for whole body changes. This high level approach can mask details about how energy is used at the biological level, similar to the joint power based approaches.

An alternate strategy is to use direct measurements of the muscle length/velocity to define the torque profile. Nuckols et al. used ultrasound measurements of the soleus velocity recorded while walking, which were quickly analysed offline (as reported in the supplemental material they captured 5s of ultrasound recording, then took 5 seconds to process images and generate torque profiles [24], orders of magnitude lower than the fastest method explored by Slade et al. [22] above), to generate a torque profile for the ankle that could then be used by the wearer. This strategy outperformed HITL-based ankle profiles in reducing metabolic cost. Further research is needed to understand why this approach outperformed prior methods.

Muscle models provide a tool for understanding how energy is used in the muscles. Hill-type muscle models [25] are a common representation, wherein the muscle-tendon-unit (MTU) is simplified to a contractile element representing the power-generating muscle fibers, a passive spring representing the tendon, and other springs and dampers in various architectures to represent the elastic and viscous properties of the MTU [26]. These models can be used to predict the torque output of the muscle based on the muscle activation level [7, 27], or, more pertinent to this paper, the energy consumption [5, 28]. The total energy rate of the muscle, $\dot{E}$, is typically estimated using a thermal model consisting of four values: the activation heat rate, $\dot{g}$, the maintenance heat rate, $\dot{h}$, the shortening heat rate, $\dot{s}$, and the mechanical work rate, $\dot{w}$, Eq 1.

$$\begin{array}{c} \dot{E}=\dot{g}+\dot{h}+\dot{s}+\dot{w} \end{array}$$ (1)

Jackson et al. [7] used this approach to examine energy consumption as a function of external ankle torques using the thermal-Hill model. They found that simply reducing muscle force does not necessarily reduce metabolic cost as it may require more work from the muscle fiber to contract a larger amount at a lower force. Metabolic cost is more significantly impacted by muscular power needs, rather than force alone, as can be seen by the $\dot{w}$ term in Eq 1.

This paper compares two proposed biologically-informed controllers for ankle exoskeletons, utilizing ultrasound data from the literature, and compares them to HITL profiles in both walking and running where available. The two proposed controllers utilize a model of adenosine triphosphate (ATP) usage from the literature [29] with the static (zero fascicle velocity) ATP hydrolysis rate removed, applied to the soleus muscle accounting for the proportion of different muscle fiber types. The static ATP hydrolisis rate was removed as we were interested in the ATP used for motion, rather than the amount used by a resting muscle. The first, force scaled (*FS*), uses the ATP model for both fast and slow twitch muscle, where *As*_*net*_ is a scale factor on the estimated muscle-tendon unit (MTU) force (*F*) converted to a torque through a conversion factor (*C*_*F*_). This scaling modifies the torque such that assistance is applied in the most ATP expensive portions of the gait cycle, i.e. when the fascicle shortening velocity is high, primarily in the second half of the stance phase. The second proposed controller, deadbanded (*DB*), applies the magnitude of a modified ATP usage model as the input to the ankle exoskeleton. The ATP model is modified with a deadband around fascicle speeds less than the speed when the tendon reaches its peak stretching velocity, limiting the potential for the exoskeleton to interfere with energy storage in the Achilles tendon, functionally shifting the curve. The peak values for each model were scaled to match the nearest speed HITL reported in the literature [8, 10] to compare for initial feasibility of the approach. These new methods for generating exoskeleton controllers based on underlying biological functions provide strong candidates for generalizable, real-time controllers which can directly transition between tasks without needing to identify the task to transition between controllers. Because these controllers use the underlying physiology they can be tailored to subjects with measurable parameters, rather than needing to optimize controller parameters. This generalizability would allow for exoskeletons to work in a wider variety of operational tasks, and opens the possibility for rapid scaling of exoskeleton distribution as time intensive, expert tuning may not be required for individual use.

## Results

### Walking

**Fig 1:**
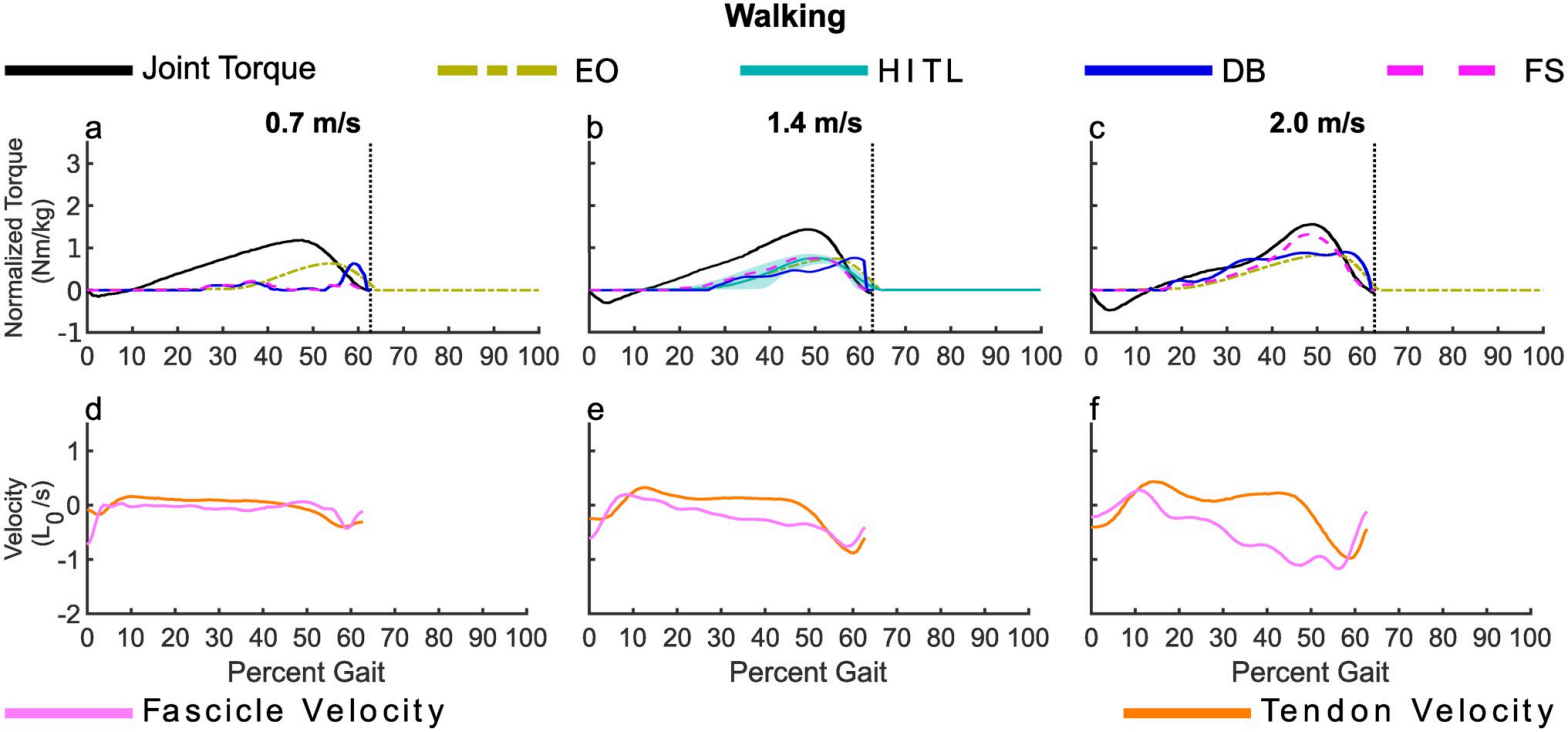
These plots show the models for walking and reference MTU dynamics. a-c) The ankle torque reported by Lai et al. [30], two methods for estimating the HITL profile using *FS* and *DB*, and the EO profile from Slade et al. [22] at the speeds matching the ultrasound data. b) For the speed with the closest match to the HITL profile speed (1.25 m/s), the HITL profile is shown for reference. It was not included at the other speeds as the HITL profile may be different for other speeds. The HITL curve is the average of the reported values from Zhang et al. [8], the shaded region is the range of the reported values. d-f) The normalized velocity of the tendon and fascicle over the gait cycle reported by Lai et al. [30].

Differences between the torque profiles derived from *FS* and *DB* (Fig 1a–1c) align with their underlying definitions. *FS* produced a torque profile of a similar shape to the human torque, as the force was the joint torque converted by a constant moment arm, then scaled by *As*_*net*_. *DB* had a shape similar to a linear ramp, mirroring the behavior of the fascicle velocity, Fig 1b. The magnitude of *FS* was more sensitive to changes in speed for walking, as shown by the greater range, [min, max], in peak values, *FS* [0.21, 1.32] Nm/kg to *DB* [0.63, 0.90] Nm/kg.

In Table 1 the range of timings reported by Zhang et al. for 1.25 m/s are presented, as well as the corresponding values for *FS* and *DB* at 1.4 m/s. From Fig 1b it can be seen that *FS* for walking speed 1.4 m/s approximates the HITL optimized torque profile for walking speed 1.25 m/s. Arnold et al. [31] found the toe-off timing difference between 1.25 and 1.5 m/s is 1% gait, so while comparisons are made at different speeds it is expected that key time points are similar and magnitude differences are accounted for by the scaling. The peak value of *FS* occurred outside of the range of reported peaks, but was within 2% percent gait of the lowest reported peak timing, and falls within the overall profile range, Fig 1b. For *DB*, the peak timing occurred later than the HITL profile. When comparing profile shapes of the proposed methods to the EO profiles, *DB* had a high cosine similarity (CS) and a low Fréchet distance (FD) when compared to *FS* at all speeds, Table 2.

**Table 1:** HITL key point range for walking at 1.25 m/s as reported by Zhang et al. [8] and the corresponding points on the different approaches, for 1.4 m/s. Bold numbers fall withing the HITL range.

	HITL Min	HITL Max	*FS*	*DB*
Time Point	Percent Gait	Percent Gait	Percent Gait	Percent Gait
Onset	17.2	37.5	**19.4**	**27.0**
Peak	48.4	52.8	46.4	58.3
Offset	59.0	64.7	**60.2**	**60.8**

**Table 2:** Profile shape comparison between the proposed controllers and an EO torque profile based on linearly interpolated HITL optimized parameters from Slade et al. [22]. The cosine similarity represents the overall similarity of profile to the EO profile, values close to 1 are more similar to the EO profile. Fréchet distance also represents similarity but is a measure of maximum distance, where smaller values are more similar.

	Walk 0.7		Walk 1.4		Walk 2.0	
	*FS*	*DB*	*FS*	*DB*	*FS*	*DB*
Cosine Similarity	0.53	0.53	0.94	0.95	0.96	0.98
Fréchet Distance	2.61	2.50	1.39	1.19	2.11	1.52

### Running

**Fig 2:**
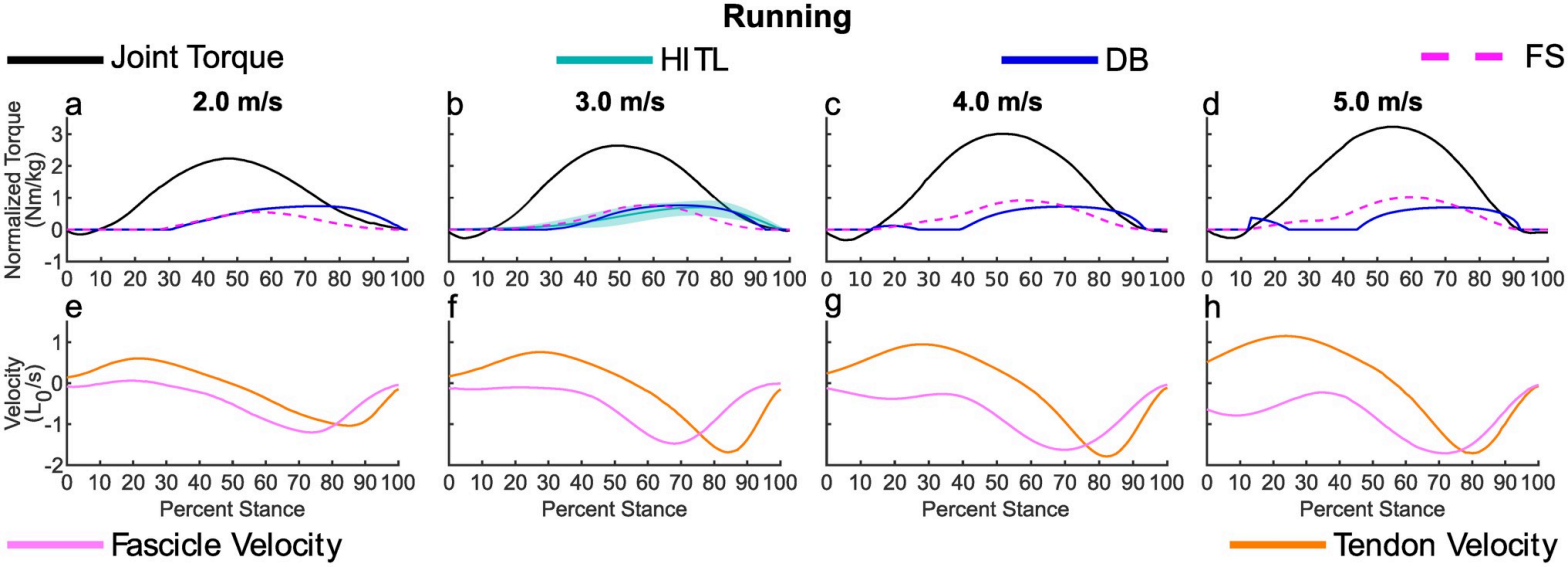
These plots show the ATP models for running and reference MTU dynamics. a-d) The ankle torque reported by Lai et al. [30], and two methods for estimating the HITL profile using *FS* and *DB*. b) For the speed with the closest match to the HITL profile speed (3.0 m/s), the HITL profile is shown for reference. It was not included at the other speeds as the HITL profile may be different for other speeds. The HITL curve is the average of the reported values from Witte et al. [10], the shaded region is the range of the reported values. e-h) The normalized velocity of the tendon and fascicle over the gait cycle reported by Lai et al. [30]. Currently, there are no EO profiles available for running.

As with walking, *FS* produced a similar profile shape to the ankle torque profile during running. As *As*_*net*_ peaks after the ankle torque similar to the fascicle velocity, the *FS* peak occurs between these two points.*DB* produced a later peak and onset than *FS* across all speeds. The magnitude of *FS* was more sensitive to changes in speed, as shown by the greater range, [min, max], in peak values *FS* [0.51, 0.93] Nm/kg to *DB* [0.64, 0.70] Nm/kg. For the two higher speeds, *DB* generated a torque application in early stance due to the bi-modal fascicle velocity, Fig 2g and 2h.

In Table 3, the range of timings for running at 3.0 m/s reported by Witte et al. [10] are presented, as well as the corresponding values for *FS* and *DB*. While onset of *FS* occurred within the range of onsets for the HITL optimized profiles, the peak and offset occurred earlier, which can also be seen in Fig 2b.*DB* was within the HITL range for both onset and peak, but offset occurred prior to the HITL range, although it was the closest method for running. The response of CS and FD for running matches that of walking, where *DB* had greater similarity, although we can only compare for a single speed in running.

**Table 3:** HITL key point range for running at 3.0 m/s as reported by Witte et al. [10] and the corresponding points on the different methods at 3.0 m/s. Bold numbers fall withing the HITL range. The cosine similarity represents the overall similarity of profile to the HITL profile, values close to 1 are more similar to the HITL profile. Fréchet distance also represents similarity but is a measure of maximum distance, where smaller values are more similar.

Time Point	HITL Min Percent Stance	HITL Max Percent Stance	*FS* Percent Stance	*DB* Percent Stance
Onset	13.6	40.5	**23**	**35**
Peak	64.5	79.5	58	**68**
Offset	93.9	98.0	86	91
Cosine Similarity	—	—	0.88	0.98
Fréchet Distance	—	—	1.92	0.74

## Discussion

This paper explored two approaches for estimating a task-invariant ankle exoskeleton torque controller based on ATP models.*DB* appears to be the strongest candidate, as it does not require any knowledge of muscle force, while producing torque profiles with similar timings to the HITL profile for both walking and running.*FS* is well suited for walking, but requires an estimate of fascicle force and does not generalize as well as the other methods, as seen by the early peak timing for running, Fig 2a–2d, andworse similarity measures for most tasks, Tables 2 and 3.

*DB* does not necessitate an estimate of the muscle force, but it does require understanding the interplay between muscle contraction and tendon loading. To benefit from the passive energy storage and return from the tendon, a low level of contraction is required so the tendon can tension. This approach used a deadband set to when the tendon was at its peak lengthening velocity, therefore limiting the amount of interference with tendon loading in early stance. However, the fascicle may lengthen (Fig 1a) or shorten (Fig 1c) at this point in the gait cycle. This simple method for setting the deadband threshold may not be effective for all scenarios and more complex methods may be needed to limit torque application in early stance. The method for applying the deadband to the ATP hydrolysis rate, while functional, is one of many possible implementations, which may be improved upon to estimate ATP hydrolysis rate beyond that needed to tension the tendon.

Time points are a convenient measure for quantifying differences between curves, but the operationally relevant difference between timings is just beginning to be studied [32]. That initial study indicates the just-noticeable-difference is around 3% gait, but it is still unclear how much these values can change before impacting performance in measures such as metabolic cost or walking speed. Because *DB* has a different profile shape than the HITL profile for walking, its peak timing occurs closer to offset and is outside of the range of HITL peaks. However, even with this different shape it was closer than FS was to EO for both CS and FD.

The shape of the HITL profile was assumed as part of the optimization process, so the HITL profiles do not necessarily represent the optimal profile shape, but rather the optimal profiles of that specified shape [8, 10]. While *DB* is different than HITL for walking, it may still be a viable candidate for this mode of locomotion. Recent work utilizing fascicle velocity during different portions of the gait cycle directly for exoskeleton control, produced a torque profile similar to *DB*, and was shown to be more effective than the HITL optimized profiles used in this paper [24]. The effectiveness of this velocity based controller can be understood to assist the most in the regions of the gait cycle where the soleus uses the most ATP, the reasoning behind the *DB* controller. The primary difference between the controllers based the ATP model and the controller used by Nuckols et al. is the hyperbola that is applied to get the ATP usage rather than the velocity directly which may result in minor differences.

Both of the methods explored here have an earlier offset than the HITL profile for running at 3.0 m/s, and may be a limitation of the single muscle model used. The gastrocnemius is a biarticular muscle (knee flexor and ankle plantarflexor). The gastrocnemius continues shortening and activating after the soleus at running speeds similar to 3.0 m/s [33]. In this way, some of the torque to flex the knee at toe-off is also used to plantarflex the ankle. Including the gastrocnemius in our model would likely increase the offset time bringing it closer to the HITL offset. An alternate explanation is that muscles do not instantaneously stop producing force [34], so they reduce their force in advance of when they should have zero force to avoid unwanted co-contractions. This limitation is one reason why exoskeleton actuation as the human joint torque decreases may be beneficial, particularly at high speeds. Further study is needed to understand how exoskeletons can be utilized in this relaxation period.

Late stance torque application may change the behavior of the fascicle, which may also impact the preferred speed of the person. The data set used for analysis had no external torque applied and at higher running speeds it is seen that there is a second mode in the fascicle velocity in early stance, Fig 2g and 2h, that creates an early stance bump in *DB*, Fig 2c and 2d. This secondary mode may be needed to generate the higher strains in the tendon for greater energy return at these increased speeds. Application of external torque in late stance may reduce the tensioning demands on the tendon and as a result reduce or eliminate this secondary mode in fascicle velocity. This reduction in fascicle velocity in early stance at high speeds may also increase preferred speed, as a reduction in fascicle velocity in this region, if it occurs, would result in a reduction in the metabolic penalty in early stance. This increase in preferred speed is observed during walking with late stance assistive torque in young adults [16, 35].

The ATP-based methods are focused mainly on the energy used by the muscle, which is important to reduce as the energy use of this muscle impacts metabolic cost. But a large portion of the ankle joint power is coming from the tendon, and balancing the contributions of these two components is an open area of exploration.

Of the methods explored in this paper, *FS* appears to be the most narrowly applicable when considering gait, limited to situations where the tendon stores less energy, like walking. For more dynamic tasks like running, where passive energy storage in the tendon becomes more important [30, 36], *FS* is expected to be less effective, also indicated by our findings. When *FS* is applied to running, the peak occurs earlier than in DB due to the MTU force occurring around mid-stance, Table 3. This method begins applying torque for running early in the stance phase, before the tendon has reached its peak lengthening velocity, Fig 2. Because of this early torque, the tendon will have less energy stored, which may require the muscle fibers to do more work in late stance, as is seen when hopping with an exoskeleton [37, 38]. So while this method may be appropriate for slower tasks, such as walking, which do not have as large tendon energy storage and return requirements, it is not well suited for tasks such as running which depend heavily on this function. Alternatively, this model may be a good candidate when applied to non-plantarflexing muscles, as the Achilles tendon serves a unique role in the human body.

### Practical considerations

This initial analysis provides insight into how modeling chemical processes in muscles can be applied in exoskeleton control and is a promising first step to creating a generalizable controller that can be applied in real time, while being adaptable to changes in activity. These methods can provide personalized assistance as they directly measure muscle fiber velocity. The direct measure accounts for the underlying physiological variability which causes differences in muscle fiber velocities that arise between different people for the same task. This section provides guidance on how to implement these controllers.

The methods proposed here will require the ability to estimate fascicle and tendon velocity, with one model also requiring force. Two possible ways to determine these values are through the use of ultrasound and through the use of machine learning. Having multiple methods for estimating MTU force and fascicle/tendon velocity allow for multiple pathways for implementing control strategies requiring force estimates. Fascicle and tendon normalized velocity can be measured using ultrasound, as was performed by Lai et al. [30]. Ultrasound has been used to understand energy usage in passive exoskeletons [39]. There has been limited use of ultrasound to modify the timing of assistive torque [40, 41], or for offline generation of torque profiles [24]. To extract fascicle length and velocity from ultrasound images for controls will require rapid extraction and processing of features from the ultrasound images, which has been demonstrated [42]. The MTU length can be calculated from the joint angle [43], and the tendon length can be found by subtracting the fascicle length.

MTU force can be estimated from ultrasound images as well [27], which has the potential for controllers based on this method to be achievable with only joint angle measurements and ultrasound images. However, if other biomechanics information (e.g. muscle activity, segment acceleration and angular velocity) is being collected for health monitoring or other purposes, these data have the potential to estimate the MTU force through the use of long-short-term-memory networks [44], or other emerging machine learning architectures. Semi-direct measurements of tendon force are also possible through shear wave tensiometer [45]. With estimated MTU force, the tendon and fascicle dynamics can also be estimated using MTU properties from the literature scaled to the wearer’s size [7].

## Limitations and future work

These models will require future human studies validation to assess the response of the controllers and wearer to each other, their effectiveness in reducing metabolic cost, and how parameters may change with different activities. While the purpose of this paper was to provide a biological basis for understanding and producing exoskeleton torque, the level of complexity and fidelity required of the models is an open question. The models used in this paper only look at a single muscle with a simplified architecture. This local view from a single muscle does not account for the influence the external torque has on the whole system, which is taken into account in HITL optimization. If these changes in the whole body impact the metabolic cost, the local approach may not be as effective, although the soleus has been shown to correlate with overall metabolic cost when external torque is applied [7]. Further study is needed to understand the effect of any new controller on the overall human-exoskeleton system.

Muscle pennation angle was not included by Lai et al. [30] and the model used assumes the MTU acts as a monolithic structure, with uniform recruitment of muscle fiber types. This assumption helps simplify the model, but does not accurately reflect muscle recruitment strategies, which first engage slow twitch muscles prior to engaging fast twitch muscles [46]. There are methods to account for the fiber recruitment strategy using EMG frequency analysis [27], but these also require more complex signal processing, which may be more challenging for real-time control. Another issue with monolithic muscle models is that MTUs are not uniform in nature. The soleus muscle spans a large portion of the shank with non uniform strain in the aponeurosis between proximal and distal portions [47]. This location-based difference in connective tissue behavior can influence fiber load transmission. Improved models that account for these differences may provide a more nuanced view of how to effectively assist the body, but controllers are possible without this more detailed understanding.

Another limitation of using data from multiple sources is that the alignment of data can create errors. Lai et al. [30] reported timing as percent stance and their reported data was converted to percent gait, which may create small timing differences for walking. The method for doing this alignment can be found in the methods section below.

Ultimately, these control strategies need to be implemented with people to understand their effectiveness and how they modify the behavior of the MTU during active exoskeleton assistance. This validation testing will also provide insight into the appropriateness of the way the profiles change with speed. The ultrasound data was collected without any external assistance, so it is unclear what changes occur in the MTU dynamics as a result of the addition of a specific external torque profile. The data comparisons between the HITL data and the data for unaided walking were collected on different individuals under different conditions. In the future, ultrasound data should be collected during HITL optimization and during application of other torque profiles, such as those proposed here, to allow analysis of individual responses to the external torque profiles. As part of this extended collection, HITL profiles should be created for a variety of speeds for both walking and running, allowing for a greater understanding of how speed influences the HITL profile. As the proposed methods generate different profiles with speed changes, having a collection of HITL profiles at different speeds would allow us to understand which of the different approaches most accurately reproduce the changes seen with changing speed. Additionally, because there are differences in timing for individuals in the HITL profiles, further study is needed to understand if these individual differences can be accounted for with the proposed methods. This validation could be completed using direct measures of the fascicle velocity or individual MTU properties to estimate the fascicle velocity. Also, the amount of error and variability in the timing of key points and magnitude of the profile should be further studied to understand the sensitivity of the metabolic cost to changes in timing and magnitude, as the sensitivity is currently unknown.

There are additional opportunities to create new controllers based on MTU dynamics [48]. The *DB* control suggests that it may be advantageous to apply torque to reduce fascicle velocity. Directly controlling the external torque to allow for moderate fascicle shortening can reduce energy usage while still allowing for tendon loading. All of the methods mentioned are areas of open exploration, but incorporating biological models into the control system has the potential to create generalizable control strategies, reducing the specialization that is currently seen in exoskeletons.

## Materials and methods

### Data sources

This paper relies entirely on data from the literature as inputs to the proposed models. The ultrasound data used for the muscle dynamics, the joint angle, and torque come from Lai et al. [30]. These normalized data were reported as the mean of the study participants (*N* = 10) and individual data was not available. The fascicle lengths and velocities were calculated from B-mode ultrasound images using automated feature extraction with manual correction when automation failed or produced poor feature identification [30]. This automated method has a coefficient of multiple correlation of 0.94 when compared to manual feature identification for prone ultrasound measurements of the medial gastrocnemius [49]. Normalization was performed on muscle fiber length, using the length for quiet standing to estimate normalized fascicle length. Tendon lengths were calculated using the difference between MTU length and the muscle fiber length accounting for pennation angle, which was in turn used to generate tendon velocity [30]. These data were used to generate the proposed exoskeleton torque profiles. The comparison HITL profiles used in this paper for walking and running come from Zhang et al. [8] (*N* = 11) and Witte et al. [10] (*N* = 12), respectively. The central HITL line is generated from the mean parameters of the study participants, the range was generated by finding curves of the max and min values across all the study participants. The EO profiles were generated using the values reported Slade et al. [22] (*N* = 3) and interpolated to find the corresponding torque profile at the speeds reported by Lai et al. These references are used for model inputs and parameters and their use is described in the following sections.

### Controllers based on ATP usage models

Muscles fibers are a collection of sarcomeres which are composed of actin and myosin fibers. When muscles contract, the heads of the myosin fiber bind to a site on the actin fiber, causing the shape of the myosin fiber head to change, pulling the actin fiber with it, at which point the actin fiber is released. To reset the shape of the myosin head, ATP is converted to adenosine diphosphate (ADP) at which point the cycle can start again [50]. Muscle fibers can be categorized by their behavior and the amount of ATPase (the enzyme that breaks down ATP) they contain [51]. Type I muscle fibers are called slow twitch as they are slower to contract, produce lower force, and have less ATPase activity. Type IIA (fast twitch) muscle are faster to contract, produce higher force, and have more ATPase activity [51, 52]. There are other muscle types but these two will be focused on in this paper.

Since ATP is used to power the muscle contraction, the amount used can estimate the energy used by the muscle, although other metabolic processes tied to ATP usage, such as ATP re-synthesis, also play a role [53, 54]. The ATP hydrolysis rate as a function of fascicle speed without static ATP hydrolysis rate can be calculated for both type I (slow twitch, low ATPase activity) and type IIA (fast twitch, high ATPase activity) muscles using Eq 2. Where *M* (the asymptotic limit to the ATP hydrolysis rate at infinite velocity) and *D* (inverse of the velocity where half the asymptotic value, *M*/2, is reached) are parameters of a hyperbola that were fit by He et al. [29] to their collected data for type I (slow) and IIA (fast) fibers. Their hyperbola has been modified by subtracting off the ATP hydrolysis rate at zero velocity, eliminating the steady state ATP hydrolysis rate so only the ATP usage for motion remains. *V*_*CE*_ is the fascicle velocity (contracti le element), *l*_0_ is the resting length during static standing, and *As* is the ATP hydrolysis rate due to fascicle velocity.

$$\begin{array}{c} As(V_{CE})=\frac{M*D*\frac{V_{CE}}{l_{0}}}{1+D*\frac{V_{CE}}{l_{0}}} \end{array}$$ (2)

This model of ATP usage was used to estimate the energy to plantarflex the ankle by the uni-articular soleus muscle. The soleus was selected as the only muscle contributing to ankle plantarflexion for generating a simplified energy profile, as it has been correlated with overall metabolic cost for walking [7, 55] and is uniarticular so it is not influenced by the motion of other joints. Reducing the energy used by the soleus by assisting in the high energy use regions of the gait cycle is expected to reduce overall metabolic cost. The ankle torque, angle, and power, as well as the normalized fascicle and tendon velocity of the soleus at various walking and running speeds were digitized from the literature [30]. This dataset only reported stance phase; for walking, the percent stance was scaled so toe-off occurred at the mean offset timing reported by Zhang et al. [8]. Stance period is related to speed in walking. Arnold et al. [31] reported walking at 1.5 m/s resulted in a 1% gait earlier toe-off timing (63±1.3% gait) compared to walking at 1.25 m/s (64±1.2% gait), which is a similar difference in speed between the ultrasound and HITL walking comparison. However offset timing reported by Zhang et al. [8] at 1.25 m/s is less than either of these times at 62.7±1.8% gait. At other walking speeds, timing event comparisons were relative within speed and would not be impacted by the selected scaling.

The original model was developed for concentric contractions where the fascicles only shortened [29] and we extended it to include the absolute value of the fascicle velocity to permit muscle lengthening in Eq 2. To account for different fiber type behavior, we estimate the net ATP hydrolysis rate for a muscle that could both lengthen and shorten with a defined composition of muscle fibers with Eq 3. The ATP hydrolysis rate from Eq 2 was calculated for the soleus for both fast and slow twitch muscles in Eq 3. Their relative contribution to the overall ATP hydrolysis rate was estimated based on the composition of the soleus muscle [28], *R*_*slow*_ = 0.8 and *R*_*fast*_ = 0.2, where R is the fraction of the muscle that is composed of slow twitch or fast twitch muscles fibers. For eccentric contractions, a correction factor of 1/2.7 was applied to account for changes in energy usage due to joint speed. The actual correction factor for the muscles fibers of the soleus may differ, as this value was experimentally determined for knee joint velocity [56]. This simplification does not directly apply to the fascicle velocity, but is taken as an approximation as fascicle-specific data for eccentric contractions were not found in the literature.

$$\begin{array}{c} \begin{array}{cc} As_{net} & (V_{CE})=\\ & \left\{ \begin{array}{cc} 0, & \text{If}\;F<0\\ \begin{array}{cc} R_{slow} & *As_{slow}(V_{CE})\\ + & R_{fast}*As_{fast}(V_{CE}), \end{array} & \text{If}\;V_{CE}\leq 0\\ \begin{array}{cc} \frac{1}{2.7}*(R_{slow} & *As_{slow}(V_{CE})\\ + & R_{fast}*As_{fast}(V_{CE})), \end{array} & \text{Otherwise} \end{array}\right. \end{array} \end{array}$$ (3)

The first model utilizing the ATP hydrolysis rate for exoskeleton control is to multiply *As*_*net*_ by the muscle force, Eq 4, creating assistance which mimics the human torque weighted by *As*_*net*_. A constant moment arm (4.2 cm) for the Achilles tendon was used to convert joint torque to muscle-tendon-unit (MTU) force, *F*. The moment arm value was selected as the mean value between 20% and 60% of the walking gait cycle, the period when the soleus is most engaged. The specific value was calculated by Rasske et al. [57] using ultrasound measurements of the Achilles tendon to determine its line of action relative to the ankle joint. The mean of the reported curve in this region of the gait cycle was used, but there was about ±2 cm standard error across subjects. There are many different models for estimating the Achilles tendon’s moment arm, but a constant moment was selected as subject-specific measurements were not available for the data sets used. As *F* is multiplied by a scale factor, using a different constant value for the moment arm would not affect the findings of this paper with respect to the shape of the torque profile, but will alter the scale factor used. The linear muscle force scaling was used as the energy consumption versus force relationship of muscles has been shown to be approximately linear [56]. As with velocity, a correction for eccentric contraction of 1/3.6 is applied for force [56]. As the eccentric contraction correction factors for torque and velocity are based on joint data and a constant moment arm was used, no additional correction is needed when applied to an MTU-based model. If a non-constant moment arm were used, the correction factors may need to be modified to account for changes in the ratio of MTU velocity to joint velocity at different angles.

$$\begin{array}{c} FS(V_{CE})=C_{F}*\{\begin{array}{cc} 0, & \text{If}\;F<0\\ F*As_{net}(V_{CE}), & \text{If}\;V_{CE}\leq 0\\ \frac{1}{3.6}*F*As_{net}(V_{CE}), & \text{Otherwise} \end{array} \end{array}$$ (4)

To scale and convert the ATP hydrolysis rate to a normalized torque, the scale factor *C*_*F*_ was defined. For both walking and running at the speed most similar to the HITL profiles from the literature [8, 10], 1.4 m/s and 3.0 m/s respectively [30], *C*_*F*_ was selected so the HITL profile and *FS* profile would have the same peak magnitude. All walking data of *FS* used the *C*_*F*_ value from the 1.4 m/s case, and all running data used the value from 3.0 m/s. This scaling was selected to allow for comparison of overall shape of the different methods at the speeds we have HITL data, but *C*_*F*_ could be made into a continuous function to account for different speeds or activities.*FS* then becomes a torque profile based on the energy used by the muscle and the MTU force.

**Fig 3:**
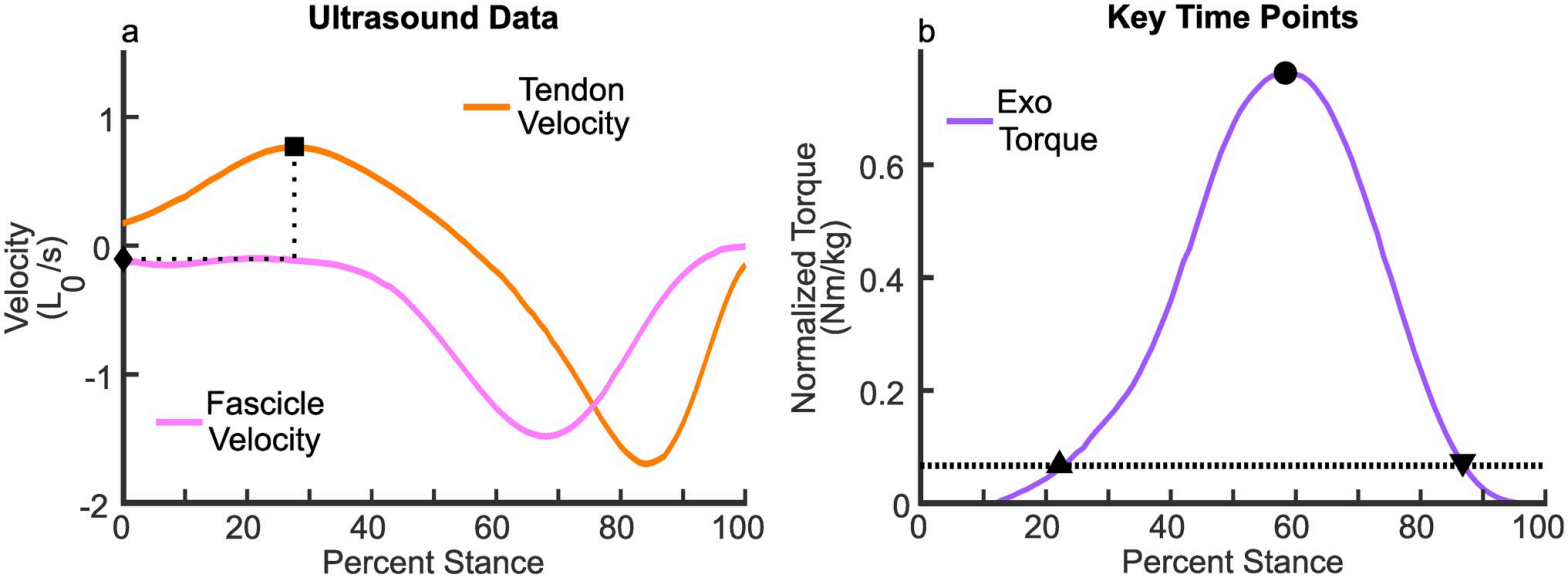
a) The fascicle and tendon velocity. $V_{CE}^{TendonVMax}$ (◆) is the fascicle velocity associated with the maximum tendon velocity (■) and was used for the deadband in Eq 5. b) A sample profile showing the onset (▲) and offset (▼) time points, which occur when the profile crosses the threshold value (dotted line). The threshold is set based on the average onset value of the HITL profile as a percent of the peak. The peak (●) corresponds to the maximum value of the profile.

To understand the advantages of using the ATP hydrolysis rate as a scale factor, as above, verses using it directly, a second model was created which uses a modified ATP hydrolysis rate. Providing external torque early in the gait cycle can interfere with tendon loading, ultimately increasing metabolic cost. Therefore, the second ATP model accounts for low-velocity contractions during tendon loading. This was not required for *FS* as in this region the joint torque is low. To this end, the fascicle velocity when the tendon reaches its maximal lengthening velocity ($V_{CE}^{TendonVMax}$), Fig 3a, was used as a deadband threshold on *V*_*CE*_, shifting the velocity so all velocities with magnitude less than or equal to $V_{CE}^{TendonVMax}$ are treated as zero velocity, Eq 5.

$$\begin{array}{c} \begin{array}{cc} As^{shifted} & (V_{CE})=\\ & \frac{M*D*\text{max}(\frac{V_{CE}}{l_{0}}-\frac{V_{CE}^{TendonVMax}}{l_{0}},0)}{1+D*\text{max}(\frac{V_{CE}}{l_{0}}-\frac{V_{CE}^{TendonVMax}}{l_{0}},0)} \end{array} \end{array}$$ (5)

As with Eq 2, the ATP usage at zero velocity is subtracted. The value of *DB* is calculated using Eq 3, with *As*^*shifted*^(*V*_*CE*_) in place of *As*(*V*_*CE*_), multiplied by a scale factor, *C*_*shifted*_, calculated in the same way as *C*_*F*_ so that *DB* and the HITL profile have the same peak magnitude for similar walking and running speeds, Eq 6.

$$\begin{array}{c} \begin{array}{cc} DB & (V_{CE})=\\ & C_{shifted}*\{\begin{array}{cc} 0, & \text{If}\;F<0\\ \begin{array}{cc} R_{slow} & *As_{slow}^{shifted}(V_{CE})\\ + & R_{fast}*As_{fast}^{shifted}(V_{CE}), \end{array} & \text{If}\;V_{CE}\leq 0\\ \begin{array}{cc} \frac{1}{2.7}*(R_{slow} & *As_{slow}^{shifted}(V_{CE})\\ + & R_{fast}*As_{fast}^{shifted}(V_{CE})), \end{array} & \text{Otherwise} \end{array} \end{array} \end{array}$$ (6)

### Comparison measures

The HITL profiles from the literature [8, 10] had three key time points: onset, peak, and offset. Prior to onset, the profile has a linear ramp to an onset torque to tension the actuation cable used in their system. At onset the torque begins to increase following a spline until it reaches its peak value, at which point it falls until it reaches offset. For the methods we explored, onset and offset torque thresholds were set based on the across subject average of the onset torque (a fixed tensioning value) as a percent of the pre-normalized peak torque (a subject-specific optimized value) of the HITL profiles. Onset for the proposed methods occurred when the rising edge of the torque profile crossed this relative percentage threshold and offset when the falling edge crossed, Fig 3b. For walking, the value 3.9% was the average percent of peak for onset reported by Zhang et al. [8]. For running, the value 8.8% was the average percent of peak for onset reported by Witte et al. [10].

Time points are important for confirming that actuation is being applied at the appropriate regions of the gait cycle, and for understanding details of the profile shape, but they don’t capture similarity of the overall shape. To evaluate overall similarity, the cosine similarity (CS) and Fréchet distance (FD) between the proposed methods and the HITL profile were calculated for the closest speed. For CS, the stance phase of the profiles were sub-sampled into 101 points (0%-100% stance, at 1% increments), these were then treated as vectors in $R^{101}$. The cosine of the angle between the two vectors is the cosine similarity measure, 1 represents identical vectors and 0 represents orthogonal vectors. CS is robust to outliers, which makes it good for comparisons where differences may occur in a small number of dimensions. It is also beneficial to have a measure of maximum error both temporally and in magnitude as brief exoskeleton actuation at an inappropriate time can be detrimental. To evaluate the magnitude of these largest differences the FD was used, which measures the largest distance between points while moving along the curves. For this measure, identical curves have a value of 0 and for curves with larger differences the value gets larger. The same 101 stance phase points were used for the FD.

## Supporting information

S1 Data Data processing. This compressed folder contains README.md, which contains instructions for running the code; UltrasoundModeling.m, a Matlab/Octave file which contains the code used to generate the plots and values found in this paper; a Digitize folder which contains comma separated variable (csv) files of the data from Lai et al. [30] digitized using WebPlotDigitizer [58]; and an empty Figures folder to store the generated plots.
(ZIP)
